# Dry Eye Subtypes in the Dry Eye Assessment and Management (DREAM) Study: A Latent Profile Analysis

**DOI:** 10.1167/tvst.11.11.13

**Published:** 2022-11-16

**Authors:** Kimberley Yu, Penny A. Asbell, Roni M. Shtein, Gui-Shuang Ying, for Dry Eye Assessment and Management Study Research Group

**Affiliations:** 1 Department of Ophthalmology, University of Southern California Roski Eye Institute, Los Angeles, CA, USA; 2 Department of Ophthalmology, University of Tennessee Health Science Center, Memphis, TN, USA; 3 Department of Ophthalmology, University of Michigan Kellogg Eye Center, Ann Arbor, MI, USA; 4 Department of Ophthalmology, Perelman School of Medicine, University of Pennsylvania, Philadelphia, PA, USA

**Keywords:** dry eye, omega-3, Sjögren's syndrome, rosacea, keratoconjunctivitis sicca

## Abstract

**Purpose:**

Dry eye disease (DED) is a heterogeneous condition with poorly characterized subtypes. The DREAM study was a large multicenter randomized clinical trial that did not find omega-3 to be more effective than placebo in treating symptomatic DED. We performed secondary analysis of DREAM data to characterize DED subtypes and their omega-3 response.

**Methods:**

A total of 535 patients with moderate-to-severe DED were randomized to omega-3 or placebo treatment for one year. We used latent profile analysis to identify subtypes based on baseline Ocular Surface Disease Index, tear break-up time (TBUT), anesthetized Schirmer's test, corneal and conjunctival staining, and meibomian gland dysfunction (MGD). We evaluated omega-3′s effect for each subtype using generalized linear regression.

**Results:**

Five clinically meaningful DED subtypes were identified. They differed significantly in sex (*P* < 0.001) and race (*P* = 0.02). Subtype 1 had the most severe DED signs yet milder symptoms and was associated with more Sjögren's syndrome (21%, *P* < 0.001). Subtype 2 had the mildest DED signs except MGD. Subtype 3 had the most severe symptoms, out of proportion to DED signs. Subtype 4 had relatively milder symptoms and MGD. Subtype 5 had severe MGD and TBUT and was associated with rosacea (29%, *P* = 0.04). Omega-3 was not significantly more beneficial than placebo for any subtype.

**Conclusions:**

Five clinically meaningful DED subtypes differed significantly in demographics, symptoms, signs, and systemic disease associations. Omega-3 was not significantly more effective than placebo for any subtype.

**Translational Relevance:**

T3 translational research identifying subtypes in the DREAM study can improve DED clinical classification and targeted management.

## Introduction

Dry eye disease (DED) is a common chronic, inflammatory, multifactorial disease of the tears and ocular surface.^1^^–^^3^ While grouped under one umbrella term, DED represents a heterogeneous group of conditions with variable underlying causes and pathophysiology that lead to tear film instability and ocular surface irritation.

Traditionally, DED is classified as aqueous tear deficient vs evaporative.^1^^,^^3^ Aqueous tear deficiency results from decreased aqueous tear production, such as seen in Sjögren's syndrome (SS). Evaporative dry eye results from any condition that increases the evaporation rate of tears, such as meibomian gland dysfunction (MGD). Irrespective of etiology, DED is also characterized by ocular surface inflammation that further exacerbates the disease.^4^^,^^5^

Although the classification of aqueous tear deficient versus evaporative dry eye is helpful, many patients have components of both aqueous tear deficient and evaporative dry eye or characteristics unexplained by either classification. The 2017 DEWS II Report suggests that DED encompasses a much more heterogeneous mixture of subtypes and causes than is captured by current classification methods.^6^ The full spectrum of DED subtypes is still poorly described, although a few recent studies have attempted to better characterize DED's heterogeneity and to define new subtypes.^7^^–^^10^

Better understanding of DED subtypes may better guide management. Current treatments for DED include artificial tears to lubricate dry eyes and prescription eye drops, such as cyclosporine and lifitegrast, to reduce ocular surface inflammation.^11^^,^^12^ These treatments alleviate symptoms and manage inflammation of DED. However, improved characterization of a patient's particular DED subtype can enable more targeted treatments and better subject selection for future clinical trials.

The Dry Eye Assessment and Management (DREAM) study was a large, multicenter randomized clinical trial of 535 adult patients with symptomatic moderate-to-severe DED to assess the efficacy and safety of an oral omega-3 supplement for the treatment of dry eye.^13^ Patients in the DREAM study underwent comprehensive evaluation of dry eye symptoms and signs following standard protocols, providing an opportunity to use the DREAM cohort to explore subtypes of DED. The DREAM study did not find omega-3 to be more effective than placebo at alleviating dry eye symptoms when all DREAM patients were considered. However, some suggest that the effect of omega-3 may differ by DED subtype.^14^^–^^16^ The aim of this study was to characterize subtypes of symptomatic dry eye in the DREAM study, and to evaluate whether omega-3 response differs among DED subtypes. Identifying DED subtypes may improve our understanding of the pathophysiology of DED, enable new subtype classifications, and guide more targeted management.

## Methods

The present study is a secondary analysis of data from the DREAM study. Full descriptions of the design and methods of the DREAM study are available in previous publications^13^^,^^17^ and at http://www.clinicaltrials.gov (identifier NCT02128763). The DREAM study was conducted in accordance to the tenets of the Declaration of Helsinki and the Health Insurance Portability and Accountability Act. The Institutional Review Boards of clinical centers participating in the DREAM study approved the study protocol and written informed consent was obtained from all patients.

### Study Patients

The DREAM study enrolled 535 adult patients with DED from 27 centers in the United States from 2014 to 2016.^13^ Patients were randomized 2:1 to receive active oral omega-3 fatty acid or placebo supplements (refined olive oil) for 12 months. The treatment group received a total daily dose of 2000 mg of eicosapentaenoic acid and 1000 mg of docosahexaenoic acid. The placebo group received a total daily dose of 5000 mg of refined olive oil (68% oleic acid, 13% palmitic acid, and 11% linoleic acid).

A broad spectrum of patients with symptomatic moderate-to-severe DED were enrolled in the DREAM study. Major eligibility criteria included age ≥18 years, a score on the Ocular Surface Disease Index (OSDI) of at least 25 to 80 at the screening visit and 21 to 80 at the baseline visit, and at least two of the following four criteria for dry eye signs in the same eye at screening and baseline visits: (1) conjunctival lissamine green staining score ≥1 (on a scale of 0 to 6); (2) corneal fluorescein staining score ≥4 (on a scale of 0 to 15); (3) tear film break up time (TBUT) ≤7 seconds; and (4) anesthetized Schirmer's test ≥1 to ≤7 mm/5 min.

### DED Symptoms and Signs

Patients underwent comprehensive evaluation of dry eye symptoms using the OSDI and dry eye signs using TBUT, Schirmer testing with anesthesia, corneal fluorescein staining, conjunctival lissamine green staining, and MGD. Following standard protocols, symptoms and signs were measured at a screening visit, baseline eligibility-confirmation visit, six-month visit, and 12-month visit.

The OSDI, determined from a 12-item questionnaire, ranges from 0 to 100 (higher scores indicate greater symptom severity). The minimal clinically meaningful change in score for an individual is 10 points.^18^^,^^19^ Dry eye signs were measured per eye. The TBUT measures time (in seconds) from a blink to the appearance of gaps in the tear film, with shorter times indicating greater tear film instability. The average of three repeated TBUT measurements was used for analysis. The Schirmer's test measures the length of wetting of paper strips placed in the inferior cul-de-sac of the lower eyelid in mm/5 minutes, with shorter lengths indicating less tear production. Corneal staining was assessed using the NEI scale whereby five areas of the cornea are graded 0 to 3, for a total possible score of 0 to 15 per eye.^20^ Conjunctival staining was assessed on a scale of 0 to 3 in the nasal and temporal areas for a total possible score of 0 to 6 per eye. Higher corneal fluorescein staining and conjunctival lissamine green staining scores indicate greater ocular surface damage. MGD was evaluated for plugging and lid secretion on a scale of 0 to 3 using the TearScience Meibomian Gland Evaluator at slit lamp, where higher scores indicate worse MGD. The average of the scores for plugging and lid secretion was used for analysis.

A composite severity score for the five dry eye signs was computed using an adapted method from previous studies.^21^^–^^24^ Each of the five signs was transformed to a common unit severity score from 0 to 1, where 0 indicates no DED and 1 indicates the most severe DED, according to the discrete severity grading system of the DEWS report.^2^ Scores between the quartile points were linearly interpolated. A composite signs severity score was calculated per eye by taking the mean of the severity scores of the five independent signs.

### Statistical Analysis to Identify DED Subtypes

To identify subtypes of DED, we performed latent profile analysis (LPA), also known as gaussian finite mixture modeling, using baseline DED symptoms—measured via OSDI—and the five individual DED signs. LPA is a modeling method that examines the underlying structure of data to identify latent (i.e., hidden or unobserved) classes (or subtypes). LPA is commonly used to classify a heterogeneous population—dry eye patients in our case—into homogenous subgroups based on similar profiles of measures (e.g., scores for dry eye symptoms and signs).^25^ To classify subtypes, LPA minimizes within-subtype variation while maximizing between-subtype variation. Posterior probabilities, which indicate the probability that a subject belongs to a given subtype, are calculated for each subtype and used to assign subjects to the subtype with the highest posterior probability.^26^ Identification of meaningful DED subtypes through LPA allows us to explore differential treatment effects across subtypes.

For each eye-specific sign, the worse eye for each patient was used for LPA. We created LPA models with one to six classes and compared their Akaike's Information Criteria and Bayesian Information Criteria for model selection. We did not test models with more than six classes to limit complexity and avoid overfitting. The DED subtypes from the three best fitting models that had the lowest Akaike's Information Criteria and Bayesian Information Criteria were presented to two dry eye specialists. The DED subtypes from the model that best represent their clinical experience managing different types of dry eye were chosen for further analysis.

The baseline demographics, systemic conditions, and DED symptoms and signs were summarized for each DED subtype. The systemic conditions evaluated were those previously found to be associated with DED severity in the DREAM study.^24^ Comparison of DED characteristics among identified DED subtypes were performed using the χ^2^ test for categorical measures and analysis of variance for continuous measures.

### Statistical Analysis to Evaluate Effect of Omega-3

We evaluated the effect of omega-3 vs placebo for each DED subtype. The outcome measures for treatment effect were the mean change from baseline in each DED symptom, sign, or composite severity score over one year. Using the same analysis method as the original DREAM study, baseline values were the means of values obtained during the screening and eligibility-confirmation visits. The values used for assessing change were the means of values obtained during the six-month and 12-month visits; if a value from only one of these visits was available, that value was used. Change in DED signs was determined for each eye separately.

Comparisons of the mean change between treatment groups and associated 95% confidence intervals (95% CI) were based on generalized linear regression with false discovery rate (FDR) adjustment for multiple comparisons. Generalized estimating equations were used to account for intereye correlations of DED signs within the same subject,^27^ performed using the R package geepack version 1.3-1.^28^

## Results

### DED Subtypes Identified Via Latent Profile Analysis

**Figure 1.**
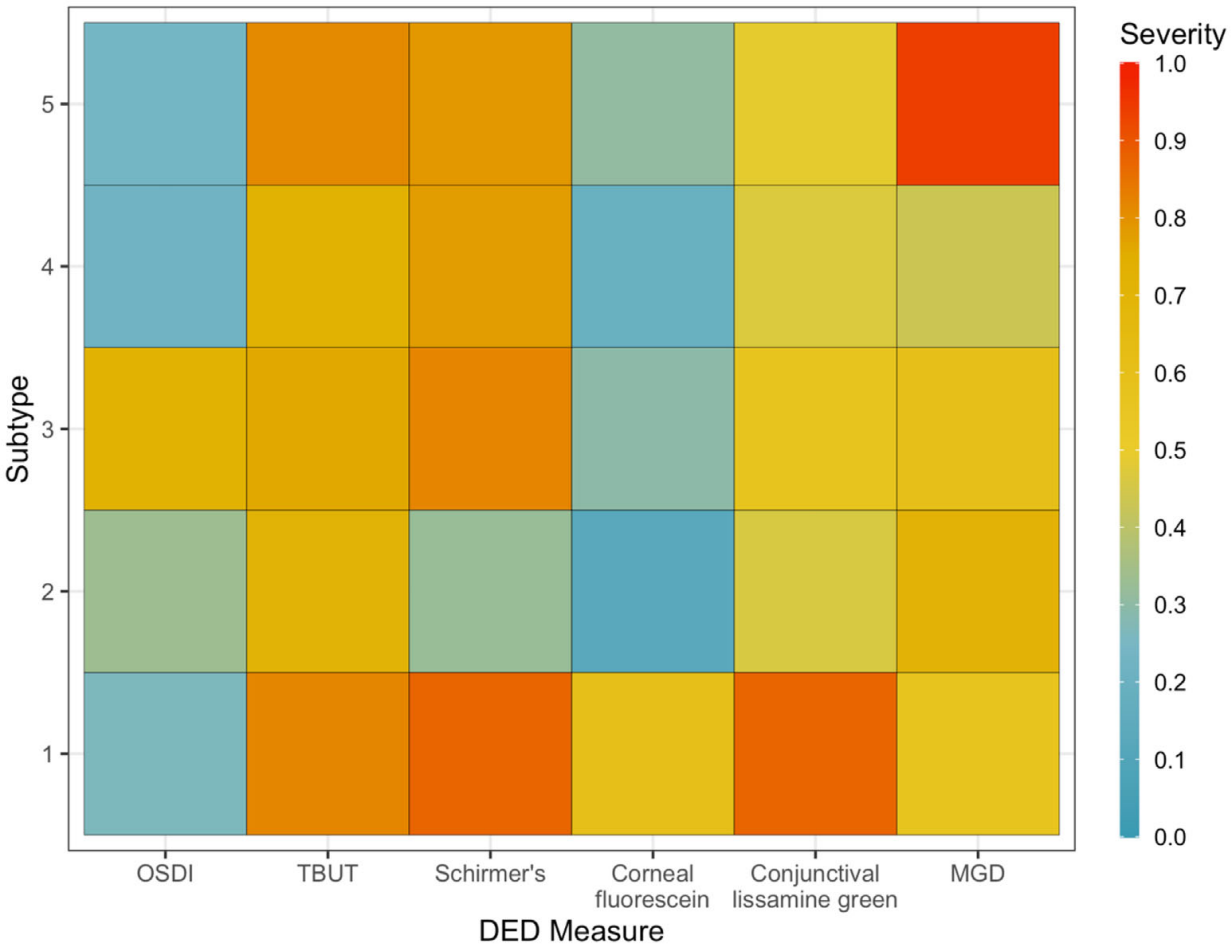
Five DED subtypes identified using latent profile analysis and consultation from two dry eye specialists. After latent profile analysis, DED signs and symptoms were transformed to a 0 to 1 scale for graphic representation, with 1 being most severe. Subtype 1 had the most severe DED signs and was significantly associated with SS. Subtype 2 had relatively milder DED signs. Subtype 3 had severe OSDI out of proportion to DED signs. Subtype 4 is the largest group likely representing a heterogenous mixture of patients. Subtype 5 had severe MGD and TBUT and was significantly associated with rosacea, consistent with evaporative dry eye.

**Figure 2.**
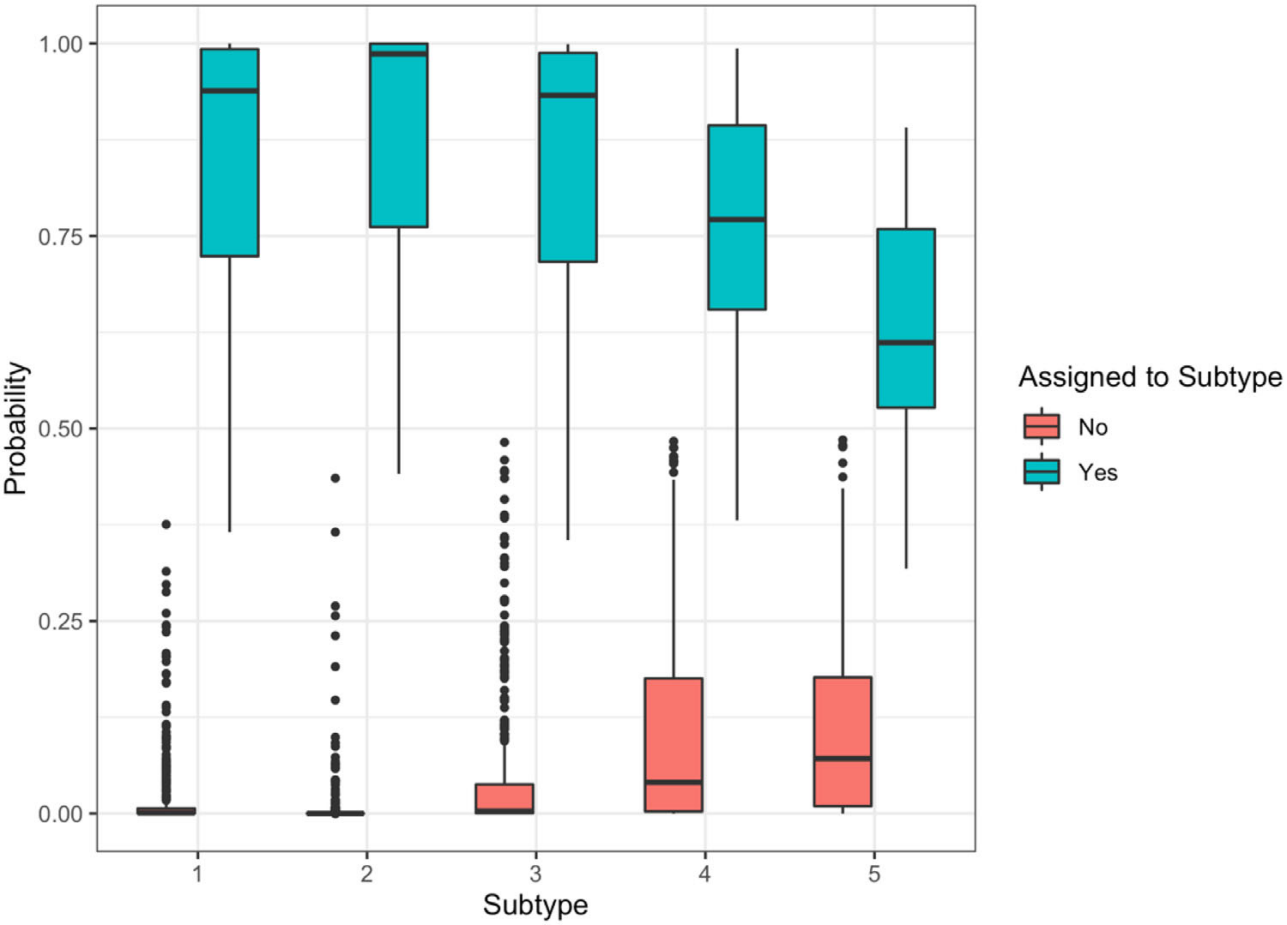
Distribution of subtype probabilities across all patients. Latent profile analysis determines the posterior probability that each patient belongs to each subtype. Patients are assigned to the subtype for which they have the highest probability. Each *dot* represents a patient, with the patient's probability of being assigned to a particular subtype plotted on the y-axis. The distribution of probabilities for patients who were assigned (*blue*) and not assigned (*red*) to each subtype are represented as *boxplots*.

Five subtypes of DED were identified through LPA and consultation with two dry eye specialists. The baseline characteristics of the 535 study patients grouped by DED subtypes are summarized in Table 1. The five subtypes had significant differences in sex (*P* < 0.001), race (*P* = 0.02), OSDI (*P* < 0.001), TBUT (*P* = 0.02), corneal staining (*P* < 0.001), conjunctival staining (*P* < 0.001), MGD (*P* = 0.03), and composite dry eye signs severity (*P* < 0.001). As shown in the heat map Figure 1 and Supplementary Figure S1, subtype 1 (89 patients) had the most severe DED signs (except MGD), yet milder OSDI. Subtype 2 (42 patients) had the mildest DED signs except MGD. Subtype 3 (119 patients) had significantly higher OSDI than the other four subtypes, with relatively average DED signs. Subtype 4 (210 patients) had relatively milder MGD and OSDI. Subtype 5 (75 patients) had the most severe MGD and severe TBUT. The distributions for the probability of assigning each patient to each subtype, as determined by LPA, are represented in Figure 2. The mean (SD) of probability of patients assigned in each subtype is 0.85 (0.18) for subtype 1, 0.88 (0.18) for subtype 2, 0.85 (0.18) for subtype 3, 0.76 (0.15) for subtype 4, and 0.63 (0.14) for subtype 5.

**Table 1.** Baseline Demographics, DED Symptoms, and Signs, and Systemic Condition Associations

Baseline Characteristics	Subtype 1 (N = 89)	Subtype 2 (N = 42)	Subtype 3 (N = 119)	Subtype 4 (N = 210)	Subtype 5 (N = 75)	*P* Value
Age (y), mean (SD)	61.1 (11.7)	50.9 (13.0)	57.6 (12.1)	57.6 (14.1)	60.2 (12.7)	0.97
Female sex (%)	81 (91.0)	26 (61.9)	101 (84.9)	163 (77.6)	63 (84.0)	<0.001
Race						0.02
White (%)	70 (78.7)	24 (57.1)	93 (78.2)	158 (75.2)	53 (70.7)	
Black (%)	6 (6.7)	6 (14.3)	10 (8.4)	29 (13.8)	13 (17.3)	
Asian (%)	7 (7.9)	2 (4.8)	2 (1.7)	6 (2.9)	2 (2.7)	
Other (%)	6 (6.7)	10 (23.8)	14 (11.8)	17 (8.1)	7 (9.3)	
OSDI score, 0–100; 100 most severe, mean (SD)	36.7 (11.1)	40.9 (14.4)	64.3 (8.5)	34.4 (8.8)	35.3 (9.6)	<0.001
TBUT, seconds; 0 most severe, mean (SD)	2.0 (0.9)	3.2 (1.1)	2.6 (1.4)	3.1 (1.5)	2.1 (0.7)	0.02
Schirmer's test, mm/5 min; 0 most severe, mean (SD)	4.4 (3.7)	23.7 (5.6)	6.3 (3.7)	7.7 (4.0)	7.5 (3.7)	0.32
Fluorescein staining of cornea, 0–15; 15 most severe, mean (SD)	9.0 (2.6)	1.9 (1.9)	4.5 (2.2)	2.9 (2.0)	4.6 (2.2)	<0.001
Lissamine green staining of conjunctiva, 0–6; 6 most severe, mean (SD)	5.2 (1.0)	2.8 (1.5)	3.4 (1.3)	2.8 (1.2)	2.9 (1.2)	<0.001
Meibomian gland dysfunction: plugging and lid secretion, 0–3; 3 most severe, mean (SD)	1.69 (0.88)	2.14 (0.86)	1.81 (0.92)	1.30 (0.70)	2.82 (0.44)	0.03
Composite dry eye severity score based on signs, 0–1; 1 most severe, mean (SD)	0.65 (0.09)	0.38 (0.08)	0.51 (0.10)	0.42 (0.07)	0.55 (0.07)	<0.001
Systemic conditions						
Sjögren's (%)	19 (21.3)	1 (2.4)	13 (10.9)	13 (6.2)	6 (8.0)	<0.001
Rheumatoid arthritis^*^ (%)	9 (10.1)	4 (9.5)	7 (5.9)	14 (6.7)	4 (5.3)	0.68
Facial rosacea (%)	17 (19.1)	5 (11.9)	31 (26.1)	34 (16.2)	22 (29.3)	0.04
Daily smoking history (%)	28 (31.5)	10 (23.8)	42 (35.3)	58 (27.6)	30 (40.0)	0.20
Peripheral artery disease (%)	13 (14.6)	1 (2.4)	8 (6.7)	14 (6.7)	11 (14.7)	0.03

* Excluding patients with secondary Sjögren's syndrome.

The five subtypes also had significant differences in the prevalence of three systemic diseases (SS, rosacea, and peripheral artery disease) previously found to be significantly associated with DED severity in the DREAM study.^24^ Subtype 1 had the highest prevalence of SS (21.3%), whereas Subtype 2 had only 2.4% SS patients (*P* < 0.001). Subtype 5 had the highest prevalence of rosacea (29.3%), whereas Subtype 2 had the lowest at 11.9% (*P* = 0.04). Prevalence of peripheral artery disease was highest for Subtypes 1 and 5 (14.6%–14.7%), while again being lowest (2.4%) for Subtype 2 (*P* = 0.03). No significant differences across subtypes were found for rheumatoid arthritis (*P* = 0.68) and history of daily smoking (*P* = 0.20).

### Effect of Omega-3 for Each DED Subtype

The difference between omega-3 and placebo groups in mean change over one year and its 95% CI within each subtype are presented in Table 2 for each DED symptom or sign. For Subtype 1 only, the mean OSDI change over one year was greater in patients receiving omega-3 (n = 60, −12.2 ± 1.9) than placebo (n = 29, −5.3 ± 1.7) (95% CI for difference: [−11.9, −1.9], FDR-adjusted *P* = 0.03). However, baseline OSDI was unbalanced between treatment and placebo groups (mean baseline OSDI of 42.3 vs 33.5, respectively; p < .001). After adjustment for baseline OSDI, the mean OSDI change was not significantly different between treatment and placebo (−11.3 ± 1.7 vs −7.1 ± 1.9, 95% CI: [−9.2, 0.8], FDR-adjusted *P* = 0.54). For all other DED subtypes, no differences between change in omega-3 versus placebo groups were significant in any of the DED symptom/sign measures (Table 2). The differences between omega-3 and placebo groups were not significant across the 5 DED subtypes in any of the DED symptoms and signs (all *P* > 0.20, Table 2).

**Table 2.** Comparison of Mean Change From Baseline Over One Year in DED Symptoms or Signs Between Omega-3 Group and Placebo Group for Five DED Subtypes

	Difference (Omega-3 Minus Placebo) in Mean Change Over One Year Between Two Treatment Groups (95% CI)					
Outcome	Subtype 1	Subtype 2	Subtype 3	Subtype 4	Subtype 5	*P* Value for Difference Across Five Subtypes
OSDI change	−6.9 (−11.9, −1.9)	−1.1 (−12.7, 10.5)	3.3 (−4.2, 10.8)	−1.2 (−6.3, 3.9)	−4.0 (−11.0, 3.0)	0.22
TBUT change (sec)	0.4 (−0.1, 0.8)	−0.2 (−1.1, 0.7)	0.1 (−0.5, 0.6)	0.1 (−0.3, 0.5)	0.2 (−0.4, 0.7)	0.90
Schirmer's change (mm/5 min)	0.5 (−0.5, 1.6)	0.0 (−3.1, 3.1)	0.9 (−0.4, 2.1)	−0.3 (−1.5, 0.9)	0.1 (−1.4, 1.5)	0.84
Corneal fluorescein score change	−0.1 (−0.8, 0.6)	0.0 (−0.6, 0.6)	0.2 (−0.3, 0.6)	0.2 (−0.1, 0.5)	−0.3 (−0.9, 0.4)	0.88
Conjunctival LG score change	0.1 (−0.2, 0.5)	0.2 (−0.3, 0.7)	−0.4 (−0.7, −0.1)	0.0 (−0.2, 0.2)	−0.1 (−0.4, 0.3)	0.36
MGD change	−0.1 (−0.3, 0.2)	0.1 (−0.2, 0.5)	0.2 (−0.1, 0.5)	−0.1 (−0.3, 0.1)	−0.2 (−0.4, 0.1)	0.28
Composite signs severity change	−0.02 (−0.05, 0.01)	0.02 (−0.02, 0.05)	0.00 (−0.03, 0.02)	0.00 (−0.02, 0.02)	−0.02 (−0.05, 0.01)	0.73

For Subtype 1, patients receiving omega-3 had a greater mean OSDI decrease over one year in comparison to patients receiving placebo (FDR-adjusted *P* value = 0.03), but this was not significant after adjustment for baseline OSDI (FDR-adjusted *P* value = 0.54). No differences between change in omega-3 versus placebo groups were significant for all other DED subtypes and DED symptom/sign measures.

## Discussion

In this study, we used latent profile analysis to identify five DED subtypes in a large, well-characterized cohort of 535 patients with moderate-to-severe dry eye disease. These subtypes differed significantly in sex, race, severity of DED symptoms and signs, and systemic disease. The differences across the five subtypes were considered clinically meaningful by dry eye specialists. We evaluated the treatment effect of omega-3 for each DED subtype and found none of the subtypes had significantly improved DED symptoms or signs after treatment with omega-3 when compared to placebo over one year.

Systemic diseases have been found to be significantly associated with DED severity,^24^ and we found notable differences in systemic disease prevalence across our identified subtypes. Subtype 1 had significantly higher prevalence of SS than the other four subtypes. SS is a systemic autoimmune disease causing severe aqueous deficient dry eye and dry mouth. Patients with SS have been found to have significantly worse TBUT, Schirmer testing, corneal staining, and conjunctival staining than non-SS DED.^29^ Despite more severe clinical signs, SS patients report less-severe subjective dry eye symptoms than non-SS dry eye patients.^30^ Congruently, Subtype 1 had the most severe DED signs, except MGD which is associated with evaporative dry eye, yet relatively milder symptoms. Subtype 1 also had a higher proportion of Caucasian women, congruent with the epidemiology of SS.^31^ We conclude that Subtype 1 represents a subtype of DED with more severe disease and reduced tear production, including but not limited to those formally diagnosed with SS. However, the severity of their dry eye may be underdetected because these patients complain less of symptoms. Attention should also be paid to any possible underlying systemic disease, like SS. For patients with increased signs of dry eyes, but not necessarily as severe of symptoms, effective DED therapy may require treating associated systemic diseases that cause or exacerbate dry eye. This subgroup may be more likely to respond well to anti-inflammatory treatments.

Subtype 2 was characterized by relatively normal to mild DED signs and the smallest proportion of patients with systemic diseases. We consider subtype 2 to represent the “traditional” dry eye group—these are patients with some signs of aqueous deficiency, some signs of evaporative concerns, and symptoms that seem to be relatively concordant to clinical signs.

Subtype 3 was characterized by severe OSDI out of proportion to DED signs. These patients may have other factors, such as neuropathic corneal pain^32^ or comorbid psychiatric conditions,^33^ that exacerbate DED symptoms.

Subtype 4 was the largest group likely representing a heterogeneous mixture of patients. Despite the power of latent profile analysis to separate and identify some of the more differentiated subtypes of patients with dry eyes, we are still left with a large proportion of patients who still fall into this heterogeneous category with no direct clinical correlations.

Subtype 5 was significantly associated with a higher prevalence of facial rosacea and, congruently, more severe evaporative signs (MGD and TBUT). Rosacea is a chronic cutaneous disorder that frequently presents with ocular signs.^34^ MGD causes alterations in meibum content leading to increased tear evaporation.^35^ Rosacea-associated MGD is a variant of MGD and is usually associated with more severe disease and inflammatory complications of the ocular surface.^34^^,^^36^^,^^37^ Patients in subtype 5 may benefit more from treatments targeting excessive evaporation, an unstable tear film, and lid hygiene.

A few other recent studies have also attempted to describe and define subtypes of DED, although our study is unique in its larger sample size and modern statistical methodology. One proposed subtype is decreased wettability due to deficient membrane-associated mucins, distinct from evaporative dry eye due to lipid layer abnormalities.^7^^,^^10^ A Delphi study classified three forms of DED according to ascending pathological severity, reversibility, and impact on visual disturbance.^8^ Other studies have focused on heterogeneity in ocular surface microbiota^9^ and tear film biomarkers^38^ to provide insights into the complicated etiology of DED.

Our classification of DED is defined by the available symptom and sign measures in the DREAM study subjects. We included the most common DED symptom and sign measures used clinically. However, these measures cannot capture finer distinctions, and do not include less commonly used measures evaluating tear film stability, tear meniscus height, lipid layer thickness, meibomian gland imaging, tear osmolarity, tear film biomarkers, and others.^39^ Further studies could include other measurements taken on DREAM subjects. Additionally, it should be noted that our study specifically identifies subtypes of moderate-to-severe symptomatic DED. Asymptomatic DED patients, such as those with poor corneal sensitivity, and symptomatic patients without clinical signs of dryness were not included in our study.^1^^,^^32^

Studies on omega-3 and its efficacy in treating DED are conflicting.^13^^,^^16^ The DREAM study did not find a significant effect of omega-3 over placebo on improving DED symptoms and signs. However, some have suggested that certain DED subtypes, such as in rosacea, blepharitis, or MGD, may respond differently.^14^^–^^16^ To further explore any potential effect of omega-3 on dry eye symptoms and signs in the DREAM study, we evaluated the effect of omega-3 in each of our identified DED subtypes. After adjustment for baseline symptoms and multiple comparisons, we did not find any significant difference in response to omega-3 versus placebo for any of the DED subtypes. These analyses for each subtype of DED are limited by small sample sizes within each subtype and unbalanced baseline symptoms and signs between omega-3 and placebo groups because of the secondary nature of our analysis.

DED is a heterogeneous condition for which the full spectrum of subtypes and underlying etiologies is still poorly understood. The current classification of dry eye is very coarse, with evaporative and aqueous-deficient dry eye being the two main groupings. The 2017 DEWS II Report emphasizes that DED encompasses a much more heterogeneous mixture of subtypes and etiologies than is captured by current classification methods.^6^ Different etiologies of dry eye are reflected by differing profiles of dry eye signs and symptoms and likely respond to different treatments with differing efficacies. More exploratory research, like that of our study, as well as others,^7^^–^^10^ are needed to better understand and describe heterogeneity within DED.

Improving classification of DED subtypes can eventually inform future clinical guidelines for more targeted and effective management of DED. Our exploratory research describing subtypes is the first step; next steps include validating our subtypes in new clinical cohorts and, if validated, developing clinical decision algorithms to classify these subtypes for easy use in clinical practice.

In conclusion, we identified five subtypes of DED that differed significantly in sex, race, severity of DED symptoms and signs, and systemic disease associations. The differences were considered clinically meaningful by dry eye specialists. The subtype associated with SS, a systemic disease known to cause aqueous tear-deficient dry eye, corresponded appropriately with worse aqueous tear-deficient DED signs. The subtype associated with facial rosacea, a disorder that can be associated with MGD, corresponded appropriately with worse evaporative DED signs. Another subtype characterized patients with severe symptoms out of proportion to signs and patients with relatively normal or mild DED signs.

## Supplementary Material

Supplement 1

Supplement 2
