# Supplementary material for: Dry Eye Subtypes in the Dry Eye Assessment and Management (DREAM) Study: A Latent Profile Analysis
Source: Transl Vis Sci Technol. 2022 Nov 16;11(11):13. doi: 10.1167/tvst.11.11.13 (PMC9680588; doi:10.1167/tvst.11.11.13)

**Supplemental Figure 1. Five DED subtypes identified using latent profile analysis and consultation from two dry eye specialists, represented as a line graph.**

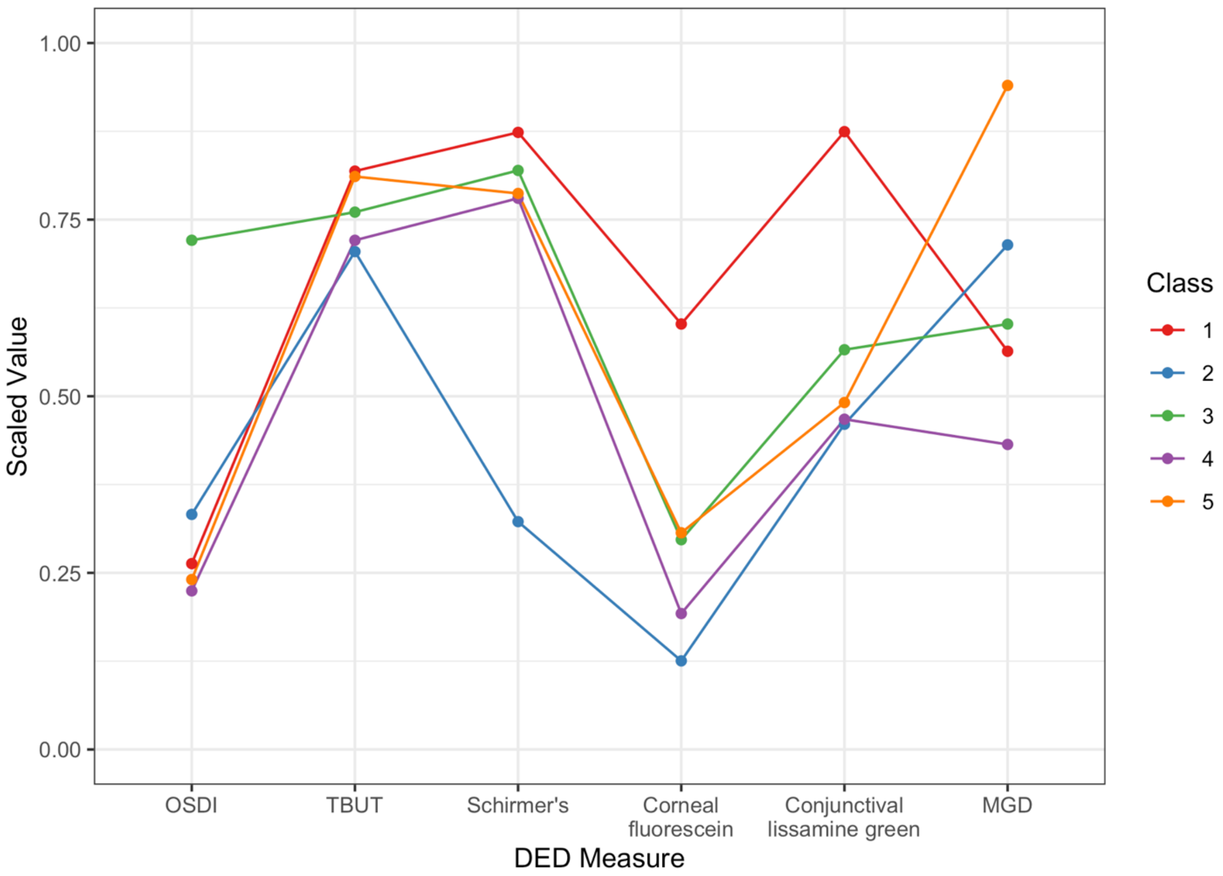

Supplement: Supplement 1 [file tvst-11-11-13_s001.pdf]
