# Supplementary material for: Dry Eye Subtypes in the Dry Eye Assessment and Management (DREAM) Study: A Latent Profile Analysis
Source: Transl Vis Sci Technol. 2022 Nov 16;11(11):13. doi: 10.1167/tvst.11.11.13 (PMC9680588; doi:10.1167/tvst.11.11.13)
Supplement: Supplement 2 [file tvst-11-11-13_s002.pdf]

Credit Roster for the DRy Eye Assessment And Management (DREAM) Study

Certified Roles at Clinical Centers: Clinician (CL); Clinic Coordinator (CC), Data Entry Staff (DE) Principal Investigator (PI), Technician (T).

Milton M. Hom (Azusa, CA): Milton M. Hom, OD FAAO (PI); Melissa Quintana (CC/T); Angela Zermeno (CC/T).

Pendleton Eye Center (Oceanside, CA): Robert Pendleton, MD, PhD. (PI); Debra McCluskey (CC); Diana Amador (T); Ivette Corona (CC/T); Victor Wechter, MD (CL).

University of California School of Optometry, Berkeley (Berkeley, CA): Meng C. Lin, OD PhD FAAO (PI); Carly Childs (CC); Uyen Do (CC); Mariel Lerma (CC); Wing Li, OD (T); Zakia Young (CC); Tiffany Yuen, OD (CC/T).

Clayton Eye Center (Morrow, GA): Harvey Dubiner, MD (PI); Heather Ambrosia, OD (C); Mary Bowser (CC/T); Peter Chen, OD (CL); Helen Dubiner, PharmD, CCRC (CC/T); Cory Fuller (CC/T); Kristen New (DE); Tu Vy Nguyen (C); Ethen Seville (CC/T); Daniel Strait, OD (CL); Christopher Wang (CC/T); Stephen Williams (CC/T); Ron Weber, MD (CL).

University of Kansas (Prairie Village, KS) John Sutphin, MD (PI); Miranda Bishara, MD (CL); Anna Bryan (CC); Asher Ertel (CC/T); Kristie Green (T); Gloria Pantoja, Ashley Small (CC); Casey Williamson (T).

Clinical Eye Research of Boston (Boston, MA): Jack Greiner, MS, OD, DO, PhD (PI); EveMarie DiPronio (CC/T); Michael Lindsay (CC/T); Andrew McPherson (CC/T); Paula Oliver (CC/T); Rina Wu (T).

Mass Eye & Ear Infirmary (Boston, MA): Reza Dana, MD (PI); Tulio Abud (T): Lauren Adams (T); Marissa Arnofsky (T); Jillian Candlish, COA (T); Pranita Chilakamarri (DE); Joseph Ciolino, MD (CL); Naomi Crandall (T); Antonio Di Zazzo (T); Merle Fernandes (T); Mansab Jafri (T); Britta Johnson (T); Ahmed Kheirkhah (T); Sally Kiebdaj (CC/T); Andrew Mullins (CC/T); Milka Nova (T); Vannarut Satitpitakul (T); Chunyi Shao (T); Kunal Suri (T); Vijeeta Tadla (CC); Saboo Ujwala (T); Jia Yin MD, PhD (T); Man Yu (T).

Kellogg Eye Center, University of Michigan (Ann Arbor, MI): Roni Shtein, MD (PI); Christopher Hood, MD (CL); Munira Hussain, MS, COA, CCRP (CC/T); Erin Manno, COT (T); Laura Rozek, COT (T/DE).

Minnesota Eye Consultants (Bloomington, MN): David R. Hardten, MD FACS (PI); Kimberly Baker (T); Alex Belsaas (T); Erich Berg (CC/T); Alyson Blakstad, OD (CL); Ken DauSchmidt (T); Lindsey Fallenstein (CC/T); Ahmad M. Fahmy OD (CL); Mona M. Fahmy OD FAAO (CL); Ginny Georges (T); Deanna E. Harter (CL); Scott G. Hauswirth, OD (CL); Madalyn Johnson (T); Ella Meshalkin (T); Rylee Pelzer (CC/T); Joshua Tisdale (CC/T); JulieAnn C. Wick (CL).

Tauber Eye Center (Kansas City, MO): Joseph Tauber, MD, PHD (PI); Megan Hefter (CC/T).

Silverstein Eye Centers (Kansas City, MO): Steven Silverstein, MD (PI); Cindy Bentley (CC/T); Eddie Dominguez (CC/T); Kelsey Kleinsasser, OD (CL).

Icahn School of Medicine at Mt. Sinai, (New York, NY): Penny Asbell, MD, FACS, MBA (PI); Brendan Barry (CC/T); Eric Kuklinski (CC/T); Afsana Amir (CC/T); Neil Chen (CC/T); Marko Oydanich (CC/T); Viola Spahiu (CC/T); An Vo, MD (T); Matthew Weinstein, DO (T).

University of Rochester Flaum Eye Institute (Rochester, NY): Tara Vaz, OD (PI); Holly Hindman, MD (PI); Rachel Aleese (CC/T); Andrea Czubinski (CC/T); Gary Gagarinas, COMT CCRA (CC/T); Peter McDowell (CC); George O'Gara (DE); Kari Steinmetz (CC/T).

University of Pennsylvania Scheie Eye Institute (Philadelphia, PA): Vatinee Bunya, MD (PI); Michael Bezzerides (CC/T); Dominique Caggiano (CC/T); Sheri Drossner (T); Joan Dupont (CC); Marybeth Keiser (CC/T); Mina Massaro, MD (CL); Stephen Orlin, MD (CL); Ryan O'Sullivan (CC/T).

Southern College of Optometry (Memphis, TN): Michael Christensen, OD PhD (PI); Havilah Adkins (CC); Randy Brafford (CC/T); Cheryl Ervin (CL); Rachel Grant OD (CL); Christina Newman (CL).

Shettle Eye Research (Largo, FL): Lee Shettle, DO (PI); Debbie Shettle (CC).

Stephen Cohen, OD, PC (Scottsdale, AZ): Stephen Cohen, OD (PI); Diane Rodman (CC/T).

Case Western Reserve University (Cleveland, OH): Loretta Szczotka-Flynn, OD PhD (PI); Tracy Caster (T); Pankaj Gupta MD MS (CL); Sangeetha Raghupathy (CC/T); Rony Sayegh, MD (CL).

Mayo Clinic Arizona (Scottsdale, AZ): Joanne Shen, MD (PI); Nora Drutz, CCRC (CC); Lauren Joyner, COA (T); Mary Mathis, COA (T); Michaele Menghini, CCRP (CC); Charlene Robinson, CCRP (CC).

Wolston & Goldberg Eye Associates (Torrance, CA): Damien Goldberg, MD (PI); Lydia Jenkins (T); Brittney Rodriguez (CC/T); Jennifer Picone Jones (CC/T); Nicole Thompson (T), Barry Wolstan, MD (CL).

Northeast Ohio Eye Surgeons (Stow, OH): Marc Jones, MD (PI); April Lemaster (CC/T); Julie Ransom-Chaney (T); William Rudy, OD (CL).

Tufts Medical Center (Boston, MA): Pedram Hamrah, MD (PI); Mildred Commodore (CC); Christian Iyore (T); Lioubov Lazarev (T): Leah Mullen (T); Nicholas Pondelis (T); Carly Satsuma (CC).

University of Illinois at Chicago (Chicago, IL): Sandeep Jain, MD (PI); Peter Cowen (CC/T); Joelle Hallak (CC);Christine Mun (CC/T); Roxana Toh (CC).

The Eye Centers of Racine & Kenosha (Racine, WI): Inder Singh, MD (PI); Pamela Lightfield (CC/T); Eunice Lowery (T); Sarita Ornelas (T); R. Krishna Sanka, MD (CL); Beth Saunders (T).

Mulqueeny Eye Centers (St. Louis, MO): Sean P. Mulqueeny, OD (PI); Maggie Pohlmeier (CC/T).

Oculus Research at Garner Eyecare Center (Raleigh, NC): Carol Aune, OD (PI); Hoda Gabriel (CC); Kim Major Walker, RN MS (CC/T); Jennifer Newsome (CC/T).

Resource Centers

Chairman's Office (Icahn School of Medicine at Mount Sinai, New York, NY): Penny Asbell, MD, FACS, MBA (Study Chair); Brendan Barry (Clinical Research Coordinator); Eric Kuklinski (Clinical Research Coordinator); Shir Levanon (Clinical Research Coordinator); Michael Farkouh, MD FRCPC, FACC, FAHA (Medical Safety Monitor); Seunghee Kim-Schulze, PhD (Consultant); Robert Chapkin, PhD, MSc. (Consultant); Giampaolo Greco, PhD (Consultant); Artemis Simopoulos, MD (Consultant); Ines Lashley (Administrative Assistant); Peter Dentone, MD (Clinical Research Coordinator); Neha Gadaria-Rathod, MD (Clinical Research Coordinator); Morgan Massingale, MS (Clinical Research Coordinator); Nataliya Antonova (Clinical Research Coordinator).

Coordinating Center (University of Pennsylvania Perelman School of Medicine, Philadelphia, PA): Maureen G. Maguire, PhD (PI); Mary Brightwell-Arnold, SCP (Systems Analyst) John Farrar, MD PhD (Consultant); Sandra Harkins (Staff Assistant); Jiayan Huang, MS (Biostatistician); Kathy McWilliams, CCRP (Protocol Monitor); Ellen Peskin, MA, CCRP (Director); Maxwell Pistilli, MS, MEd (Biostatistician); Susan Ryan (Financial Administrator); Hilary Smolen (Research Fellow); Claressa Whearry (Administrative Coordinator); Gui-Shuang Ying, PhD (Senior Biostatistician) Yinxi Yu (Biostatistician).

Biomarker Laboratory (Icahn School of Medicine at Mount Sinai, New York, NY): Yi Wei, PhD, DVM (co-Director, Biomarker Laboratory); Neeta Roy, PhD (co-Director, Biomarker Laboratory); Seth Epstein, MD (Former co-Director; Biomarker Laboratory); Penny A. Asbell, MD, FACS, MBA (Director and Study Chair).

Investigational Drug Service (University of Pennsylvania Perelman School of Medicine, Philadelphia, PA): Kenneth Rockwell, Jr., PharmD MS (Director).

Peroxisomal Diseases Laboratory at the Kennedy Krieger Institute, Johns Hopkins University Baltimore MD: Ann Moser (Co-Director/Consultant); Richard O. Jones, PhD (Co-Director/Consultant)

Meibomian Gland Reading Center (University of Pennsylvania Perelman School of Medicine, Philadelphia, PA): Ebenezer Daniel, MBBS, MPH, PhD, (PI); E. Revell Martin (Image Grader); Candace Parker Ostroff, (Image Grader); Eli Smith (Image Grader); Pooja Axay Kadakia (Student Researcher).

National Eye Institute, National Institutes of Health, Department of Health and Human Services: Maryann Redford, DDS, MPH (Program Officer).

Office of Dietary Supplements/National Institutes of Health, Department of Health and Human Services

Committees

Executive Committee (Members from all terms of appointment): Penny Asbell, MD FACS, MBA (Chair); Brendan Barry, MS; Munira Hussain, MS, COA, CCRP; Jack Greiner, MS, OD, DO, PhD; Milton Hom, OD, FAAO; Holly Hindman, MD, MPH; Eric Kuklinski, BA; Meng C. Lin OD, PhD. FAAO; Maureen G. Maguire, PhD; Kathy McWilliams, CCRP; Ellen Peskin, MA, CCRP; Maryann Redford, DDS, MPH; Roni Shtein, MD, MS; Steven Silverstein, MD; John Sutphin, MD.

Operations Committee: Penny Asbell, MD FACS, MBA (Chair); Brendan Barry, MS; Eric Kuklinski, BA; Maureen G. Maguire, PhD; Kathleen McWilliams, CCRP, Ellen Peskin, MA, CCRP; Maryann Redford, DDS, MPH.

Clinic Monitoring Committee: Ellen Peskin, MA, CCRP (Chair); Mary Brightwell-Arnold, SCP, Maureen G. Maguire, PhD; Kathleen McWilliams, CCRP.

Data and Safety Monitoring Committee: Stephen Wisniewski, PhD (Chair); Tom Brenna, PhD; William G. Christen Jr, SCD, OD, PhD; Jin-Feng Huang, PhD; Cynthia S. McCarthy, DHCE, MA; Susan T. Mayne, PhD; Mari Palta, PhD; Oliver D. Schein, MD, MPH, MBA.

Industry Contributors of Products and Services

Access Business Group, LLC (Ada, MI) Jennifer Chuang, PhD. CCRP; Maydee Marchan, M.Ch.E; Tian Hao, PhD; Christine Heisler; Charles Hu, PhD; Clint Throop, Vikas Moolchandani, PhD.

Compounded Solutions in Pharmacy (Monroe, CT)

Leiter's (San Jose, CA)

Immco Diagnostics Inc. (Buffalo NY

OCULUS Inc. (Arlington, WA)

RPS Diagnostics, Inc. (Sarasota, FL)

TearLab Corporation (San Diego, CA)

TearScience Inc. (Morrisville, NC)
